# Dietary Intakes of Minerals, Essential and Toxic Trace Elements for Adults from *Eragrostis tef* L.: A Nutritional Assessment

**DOI:** 10.3390/nu10040479

**Published:** 2018-04-12

**Authors:** Eva Koubová, Daniela Sumczynski, Lenka Šenkárová, Jana Orsavová, Miroslav Fišera

**Affiliations:** 1 Department of Food Analysis and Chemistry, Tomas Bata University in Zlín, Náměstí T.G. Masaryka 5555, 760 01 Zlín, Czech Republic; kotaskova@ft.utb.cz (E.K.); fisera@utb.cz (M.F.); 2 Department of Environmental Protection Engineering, Tomas Bata University in Zlín, Náměstí T.G. Masaryka 5555, 760 01 Zlín, Czech Republic; lveverkova@ft.utb.cz; 3 Language Centre, Tomas Bata University in Zlín, Štefánikova 5670, 760 01 Zlín, Czech Republic; orsavova@fhs.utb.cz

**Keywords:** *Eragrostis tef*, essential trace elements, toxic trace elements, minerals, dietary intakes, ICP-MS

## Abstract

This study analysed the contents of thirty-six mineral and trace elements in teff (*Eragrostis tef* L.) grains. What is more, dietary intakes were calculated. Inductively coupled plasma mass spectrometry (ICP-MS) was used to assess mineral and trace element contents. Consequently, the appropriate Recommended Dietary Allowance (RDA) or adequate intake (AI), and provisional tolerable weekly intake (PTWI) or provisional tolerable monthly intake (PTMI) values for adults were determined according to the Food and Agriculture Organization/World Health Organization (FAO/WHO) and Institute of Medicine (IOM) regulations. Teff is a significant contributor to RDAs and AIs for females in the following order: Mn > Cu > Zn ≥ Mg > Fe ≥ P and Ca. For males, teff contributes in the order, Mn > Cu > Fe > Zn ≥ P ≥ Mg > and Ca. The concentration of arsenic (65.9 µg/kg) in brown teff originating in Bolivia exceeded the average acceptable value set by Reg. No. 1881 of 6–50 µg/kg in cereals consumed in the EU. The PTWIs or PTMIs for Al, Cd, Sn and Hg were all under 7%, which is below the limits of toxic element intake related to the body weight of 65 kg for adult females and 80 kg for males, set by the FAO/WHO. Teff grains can be recommended as a valuable and safe source of minerals and trace elements.

## 1. Introduction

Value-added foods provide health benefits above normal nutrition. Dietary fibre, vitamins, minerals and antioxidants enhance health in general. Nowadays, interest in the utilization of alternative grains in food production has been growing [1] due to consumers’ demand for food promoting health and well-being. The awareness of toxic and potentially harmful substances has been rising as well.

Teff (*Eragrostis tef* L.), originating from Ethiopia, is commonly used in flat bread production [2]. Unfortunately, only scarce research has been conducted to examine teff’s potential and its rich nutritional benefits. Two types of teff are distinguished according to seed colour—white or brown—both belonging to the millet group [3,4]. Currently, Dutch markets have a focus on growing teff [5]. However, as only a small quantity of teff grown in Europe actually appears in local markets, most teff is still imported from Bolivia.

Trace and toxic elements have attracted public attention regarding their possible impact on health. However, their dietary intakes still need to be monitored to establish their safety in foods. Inorganic contaminants, such as As, Cd, Pb, Sn, Al and Hg, are the most studied toxic elements. The Joint Food and Agriculture Organization/World Health Organization (FAO/WHO) Committee on Food Additives (JECFA) has set the values of provisional tolerable weekly (PTWI) and monthly intakes (PTMI), as well as establishing further contaminants in food [6]. Teff is a valuable source of minerals; in particular, Ca, Fe, Mn and Zn are present in larger amounts. Since teff is consumed as a whole grain, it may provide desirable nutrients and bioactive compounds. Unfortunately, literature data focusing on particular trace and toxic elements in teff and data evaluating the contribution of teff grains to the RDA (Recommended Dietary Allowance), AI (Adequate intake), PTWI and PTMI values has been scarce.

Consequently, this study was conducted to determine the content of minerals (Na, P, K, Mg and Ca) and trace elements (Mn, Zn, Fe, Cu, Be, Ni, Al, Ga, Co., Li, Sc, Ag, Sr, Ba, Tl, Bi, Ce, Cs, Ho, Ta, Tb, U, Y, Cr, Se, Sn, As, Pb, Hg, Cd and Ti) in brown and white teff grains by Inductively coupled plasma mass spectrometry(ICP-MS) and to establish their contributions to RDA, AI, PTWI and PTMI values.

## 2. Materials and Methods

### 2.1. Grain Sample Preparation and Reagents

Samples of *Eragrostis tef* L. were prepared from white and brown grains from Bolivia, harvested in 2016 and 2017, and from brown grains produced in the EU in 2017. European and Bolivian grains were bought at local markets in the Czech Republic, each as five packages of 250 g. Teff samples originating in the USA were bought at local markets in Idaho—five packages of 450 g. The samples were stored in dark air-lid plastic boxes. Each sample was analysed five times.

ICP-MS STANDARD 13 standard series (As, Ca, Cd, Cr, Fe, Hg, K, P, Na, Pb, Se, Sn and Ti), ICP-MS STANDARD 23 standard series (Be, Zn, Cu, Ni, Al, Ga, Mg, Co., Li, Sc, Ag, Mn, Sr, Ba, TI, Bi, Ce, Cs, Ho, Ta, Tb, U, Y), ICP-MS INT Rh (Analytika Ltd., Czech Republic), Analpure ultra H_2_O_2_ and 67% Analpure ultra HNO_3_ were purchased from Analytika (Prague, Czech Republic). Helium and argon were obtained from Linde Gas (Zlín, Czech Republic), and ultrapure water was supplied by Purelab Classic Elga water system (Labwater/VWS Ltd., London, UK).

### 2.2. ICP-MS Analysis

#### 2.2.1. Sample Preparation

High purity 18.2 MΩcm water was obtained from Purelab Classic Elga system. Five millilitres of 67% HNO_3_ and 1 mL of H_2_O_2_ were added to each sample, resulting in sample weights of 1.0 ± 0.0001 g. Then they were decomposed by a microwave system, Milestone Ethos One (Sorisole, Italy), set to the following parameters: 500 W for 10 min, 1500 W for 15 min and finally, 500 W for 15 min. Two sets of calibration standard series were prepared to be matched with the expected concentration ranges in the samples: a high standard series (23 elements) (^9^Be, ^66^Zn, ^63^Cu, ^60^Ni, ^27^Al, ^71^Ga, ^24^Mg, ^59^Co, ^7^Li, ^45^Sc, ^107^Ag, ^55^Mn, ^88^Sr, ^137^Ba, ^205^Tl, ^209^Bi, ^140^Ce, ^133^Cs, ^165^Ho, ^181^Ta, ^159^Tb, ^238^U and ^89^Y) at concentrations of 3–35 µg/l and a low standard series (13 elements) (^75^As, ^44^Ca, ^111^Cd, ^52^Cr, ^57^Fe, ^202^Hg, ^39^K, ^31^P, ^23^Na, ^208^Pb, ^77^Se, ^118^Sn and ^48^Ti) with concentrations between 0.5 and 1.0 µg/L. Rhodium ^103^Rh, at a concentration of 100 µg/L, was used as an internal standard. Statistical evaluation was performed by certified reference material analysis (CRM) of green algae Metranal^®^8 (Analytica Ltd., Jílové, Czech Republic) producing values in mg/kg for As (41 ± 3), Ca (1380 ± 80), Cd (0.023 ± 0.004), Co. (18.0 ± 1.6), Cu (34,0 ± 1.6), Fe (290 ± 20), Hg (0.017 ± 0.010), Mg (1580 ± 120), Mn (43.0 ± 3.4), Ni (0.8 ± 0,1), Pb (0.21 ± 0.01) and Zn (38 ± 3).

#### 2.2.2. ICP-MS Instrumentation

Analyses were performed by a quadrupole-based Thermo Scientific iCAP Qc inductively coupled plasma-mass spectrometer (ICP-MS) (Thermo Scientific, MA, USA). Furthermore, a collision cell (QCell) containing helium was applied to remove undesirable molecule ions by distinguishing their kinetic energy (CCT, collision cell technology; KED, kinetic energy discrimination mode). Specific working parameters were set as follows: 1550 W power, sampling depth of 5-mm, cool gas flow rate of 14.0 L/min, auxiliary gas flow rate of 0.8 L/min, nebulizer gas flow rate of 1.015 L/min, He flow rate of 4.1 mL/min, nebulizer pump speed of 40.00 rpm and chamber temperature of 2.7 °C. Samples were analysed five times.

### 2.3. Evaluation of Minerals Contribution to the RDA, AI, PTWI and PTMI Values

Dietary intake levels for nutrients from teff were established and compared with the RDA or AI (if the RDA had not been set) as recommended by the IOM (Institute of Medicine) [7,8,9,10]. Intake levels of toxic elements were also estimated and compared with the PTWI or PTMI (if the PTWI had not been established) recommendations [11,12,13,14]. Since there is no recommendation for the daily intake of teff grains, a portion size of teff was set to 100 g. Intake levels were determined for both females and males aged between 31 and 50, for males weighing 80 kg and females weighing 65 kg.

### 2.4. Statistical Analysis

All analyses were repeated 5 times (*n* = 5). The results are reported as the mean ± standard deviation (SD) in fresh weight and were statistically evaluated by one-way analysis of variance (ANOVA). Subsequently, the Scheffe test was applied to identify differences between all possible pairs of samples (multiple comparisons). The level of significance was set to 0.05.

## 3. Results and Discussions

Nine teff samples were examined for thirty-six elements. Table 1 summarises the analytical data according to their amount (mg/kg or μg/kg) in fresh weight. Because of a relatively low number of representative brown and white grains, differences between these two grain types cannot be discussed.

### 3.1. Essential Macronutrient Measurement

The highest sodium content was recorded in teff from Bolivia (185 mg/kg). Except for the Bolivian samples, the results of this study were in accordance with the USDA (United States Department of Agriculture) [15] data (120 mg/kg). Sodium plays an important role in the regulation of blood pressure. The AI value for Na has been set at 1500 mg/day for both females and males aged between 31 and 50 [10].

The magnesium concentration in teff ranged from 1760 to 2530 mg/kg. According to the USDA [15], teff contains 1840 mg/kg of magnesium. Interestingly, magnesium content in teff was five or six times higher than in wheat [16]. Symptoms of magnesium deficiency can include muscle weakness and cardiac malfunction. The RDA values for females and males have been established at 320 and 420 mg/day, respectively [7].

Phosphorus content in teff reached 4180 mg/kg. According to the USDA [15], teff may contain 4300 mg/kg of phosphorus. The results in this study were below this limit. Phosphorus is stored in the form of phytic acid which is an antinutrient due to its affinity for Fe, Zn and Ca [17]. Cells employ phosphate in the transport of cellular energy in the form of adenosine triphosphate, and together with calcium salts, it assists in stiffening bones. The RDA value for females and males aged 31‒50 years is 700 mg/day [7].

According to the USDA [15], the potassium content in teff is 4270 mg/kg. In this study, the highest potassium content was found in brown teff from Bolivia (4290 and 4730 mg/kg). Potassium is an intracellular cation that acts as a regulator in cellular osmotic balance [18]. The AI value for females and males aged 31‒50 is 4700 mg/day [10].

Calcium content ranged from 1650 to 2650 mg/kg. According to the USDA [15], teff contains 1800 mg/kg calcium. Abebe et al. [17] reported the calcium content in teff to vary between 1200 and 1500 mg/kg. In this study, the samples contained higher calcium contents. Calcium, stored in the bones, is essential for muscle contraction and hormone secretion. The RDA value for females and males aged 31‒50 is 1000 mg/day [7].

### 3.2. Essential Trace Elements Measurement

It is known that trivalent chromium enhances insulin activity and participates in the metabolism of carbohydrates. However, hexavalent chromium is toxic [6]. The chromium content in teff ranged between 2.3 and 23.2 µg/kg. Kabata-Pendias [19] reported the chromium level in cereals as varying from 10 to 90 µg/kg. The AIs for males and females aged 31‒50 are 35 and 25 µg/day, respectively [9].

Manganese concentrations in teff were between 17.1 and 68.4 mg/kg. The USDA [15] determined the manganese content in teff to be about 92.4 mg/kg. Our results did not reach the USDA limit. Manganese may function as an enzyme activator for several enzymes. Due to its ubiquity in food, deficiency is improbable if an adequate diet is kept [20]. The IOM [9] has set the AI values for manganese for males and females aged 31‒50 as 2.3 and 1.8 mg/day, respectively.

Most of the amount of iron in the body is found in heme proteins. Generally, the amount of available iron in staple plants is low due to the presence of phytic acid. The highest iron content was detected in teff from the USA, ranging from 111 to 117 mg/kg. Abebe et al. [17] reported an iron content in brown teff of over 150 mg/kg and in white teff of 37.7 mg/kg. The RDA values for females and males are 18 and 8 mg/day, respectively [9].

The copper content in teff ranged between 6.12 and 25.30 mg/kg. The USDA [15] declared teff to have a copper content of 8.1 mg/kg, which is comparable with the results of teff originating in Bolivia. Copper in is essential for the human body in small quantities as a constituent of redox enzymes. The RDA value for copper is 900 µg/day for both females and males aged 31‒50 [9].

The highest zinc levels were detected in teff from the USA in amounts between 66.9 and 74.2 mg/kg. The USDA [15] declared the zinc content in teff to be 36.3 mg/kg; Abebe et al. [17] reported the zinc content in brown teff as about 40.2 mg/kg and 28.6 mg/kg in white teff. As can be seen from the results, teff originating in the USA contained almost twice as much zinc as the quantities published in the studies mentioned above. The high zinc concentrations found in teff may eliminate zinc deficiency in a diet, but the chelating effect of phytic acid has to be considered. The RDA values for females and males aged 31‒50 are 8 and 11 mg/day, respectively [9].

The selenium content in teff grains was ranged from 8.61 to 19.30 µg/kg, with lower selenium concentrations determined in teff from Bolivia. Hongxing and Yu-Kui [21] did not find any selenium in millet. In contrast, Orecchio et al. [22] declared the selenium content in gluten-free flour to be 72 µg/kg. The selenium content in crops has recently received much attention. Selenium is recognized as a cellular antioxidant and protective agent against several toxic elements [20]. The RDA value for females and males aged 1‒50 years is 55 µg/day [8].

The cobalt content ranged from 25.9 to 42.1 µg/kg. Neither the RDA nor the AI levels have been determined yet.

### 3.3. Toxic Trace Element Measurement

The potential toxicity of aluminium is connected with neurotoxicity [20]. Several studies have referred to a link between Alzheimer’s disease and aluminium, but a causal relationship has not been established yet [23]. The lowest and highest aluminium contents in teff were 5.42 and 13.40 mg/kg, respectively. As a comparison with other cereals, the aluminium content in rice, as determined by Millour et al. [24] is only 1.5 mg/kg. The PTWI value for aluminium is 2 mg/kg bw [13].

The average arsenic content in cereals consumed in the EU varies between 6 and 50 µg/kg [25]. Arsenic content in brown teff from Bolivia harvested in 2017 exceeded these values. Antoine et al. [18] detected arsenic content in rice in the range of 82–487 µg/kg. Arsenic may be toxic, and its toxicity varies with its chemical form. Inorganic compounds are toxic, while organic compounds show only a low toxicity [20]. Exposure to arsenic may cause dermatitis or may even have carcinogenic effects. The JECFA committee has reported that the current PTWI values for arsenic of 2.1 µg/kg bw per day are not health protective if the BMDL 0.5 value (benchmark dose lower confidence limit for 0.5% increased incidence of lung cancer in humans) is in the same range as the PTWI. Therefore, the PTWI for arsenic has been withdrawn [12].

Cadmium is a nephrotoxin that accumulates in the human body and thus, should be monitored. The PTMI value is 25 µg/kg bw [14]. The average level of cadmium content in cereals consumed in the EU varies from 3.8 to 58.4 µg/kg, and the maximal cadmium content in cereals has been established at 100 µg/kg [26]. As can be seen from the results of this study, cadmium concentrations found in teff did not reach this limit.

The tin content in teff was between 0.50 and 1.72 µg/kg. Millour et al. [27] published 5.0 µg/kg of tin in cereals. Tin is a less toxic element. Even though the half-life of tin is substantially long, its accumulation in tissues is limited due to its rapid movement through the gastrointestinal tract. However, tin may cause acute gastrointestinal tract issues, such as abdominal distension and vomiting, presumably if it is ingested in high doses. The PTWI for tin is 14 mg/kg bw [11,28].

Barium amounts ranged between 2.21 and 7.02 mg/kg. These results are in accordance with published data.

The toxic effects of mercury compounds are generally well-known. Symptoms of poisoning include neurological and cardiovascular diseases [6]. Therefore, a PTWI value of 4 µg/kg bw has been determined [12]. According to [25], the average mercury level in cereals consumed in the EU is between 2.7 and 19.0 µg/kg. However, the maximal mercury level in cereal products has not been defined yet. The range of mercury contents in teff was from 1.42 to 3.11 µg/kg, which can be considered to be insignificant.

Even though lead is harmful to the nervous system and causes blood disorders [6], the PTWI value for lead (25 µg/kg bw) was withdrawn in 2011 [29]. Reg. No. 420 [30] defines the maximal lead level in cereals as 200 µg/kg and the average level in food ranges from 5 to 139 µg/kg [25]. In this study, low lead concentrations in teff were recorded.

The highest bismuth concentrations were detected in brown teff from Bolivia. Gonzáles-Weller et al. [31] reported bismuth contents in cereal products reaching 1700 µg/kg. In contrast, Matos-Reyes et al. [32] did not detect bismuth in rice at all. Bismuth toxicity was reported after exposure during the therapeutic treatment affected livers and kidneys [20].

### 3.4. Measurement of Other Trace Elements 

As far as lithium is concerned, the highest amount was detected in teff from the USA. Kabata-Pendias [19] reported a lithium content in the *Gramineae* family grown in the USA between 70 and 1500 µg/kg, and Hongxing and Yu-Kui [21] reported a lithium content in millet of 12 µg/kg.

Relevant data discussing beryllium, scandium and titanium contents in millet has been scarce. Generally, beryllium is found in plant samples at concentrations of 0.1–25.0 µg/kg [19]. Nyarko et al. [33] reported the scandium content in rice as 91.3 µg/kg, and the content of titanium in rice varies considerably within the range of 410–1390 µg/kg [18]. These results are in accordance with the previously mentioned studies. In this analysis, the lowest concentrations of nickel were recorded in teff from Bolivia. Noël et al. [34] reported the nickel content in cereals to be 182–280µg/kg, and Kabata-Pendias [19] stated that the nickel content in cereal grains ranges from 340 to 1280 µg/kg. Nickel is a carcinogenic metal documented to initiate epigenetic alteration of a cell into a cancer one. Therefore, it is important to monitor nickel concentrations in food.

According to Kabata-Pendias [19], gallium has been commonly recorded in plant tissues, and its concentration varies from 0.02 to 30.00 mg/kg. However, detailed data focusing on gallium content in cereals has been limited.

Millour et al. [27] reported a strontium content in cereals of 1830 µg/kg, and strontium in teff grains was recorded between 215 and 527 µg/kg. Higher concentrations of strontium seem to impair regular bone development.

The content of yttrium in teff reached 34.7 µg/kg. Data considering the amount of yttrium in cereals is scarce. Kabata-Pendias [19] stated that the yttrium content can be up to 3.50 mg/kg in cereals depending on soil and climatic factors. Yttrium may be absorbed by plants and subsequently enter human bodies through the linkages of food chains.

The content of silver in teff was monitored between 13.4 and 34.9 µg/kg which is less than the 42–84 µg/kg reported in cereals by Millour et al. [27]. The natural biological functions of silver in the human body and the possible health effects of silver have not been described yet.

Due to limited information about caesium and cerium in teff, results from the current study were compared with the study provided by Antoine et al. [18], who determined the caesium content in rice to be in the range of 3–110 µg/kg and the cerium content to be in the range of 20–100 µg/kg.

Terbium is one of the rare earth elements included in the estimation of toxic effects on plants; therefore, it may serve as a representative element to examine the extent of pollution [35]. Even though data focusing on terbium content has been scarce, Kabata-Pendias [19] reported its average content in mushrooms as 0.8–1.6 µg/kg.

Albeit plants do not absorb holmium from the soil, it may accumulate in teff in low concentrations.

In this study, tantalum was detected only in brown types of teff. Kabata-Pendias [19] reported its content in cereals as1.1–5.0 µg/kg.

Thallium values were between 1.10 and 2.42 µg/kg, which is low in comparison with data published by Xiao et al. [36], who recorded the thallium content in edible cereals as ranging from 30 to 300 µg/kg.

Starch-rich foods, such as seeds and flour, have been proven to be uranium poor. Antoine et al. [18] determined the minor uranium content in rice as between 1 and 20 µg/kg, while Anke et al. [37] reported its content in oat flakes as 1200 µg/kg. The amount of uranium teff was very low.

### 3.5. Estimation of Dietary Intakes of Minerals and Essential Trace Elements from Teff Grains

Dietary intakes of essential minerals and trace elements in teff were estimated and compared with the RDA or AI values for adults aged 31−50 (Table 2).

It can be observed that teff contributes insignificantly to the AIs of sodium, potassium and chromium, and to RDA of selenium. This should be applied in the recommendations for consumers. It is beneficial to obtain less than 1% of the AI value for sodium to prevent hypertension. Teff grains provide 7% of the AI for chromium for males and 9% for females; and also 10% of the AI for potassium for both males and females. Teff is not an efficient source of selenium (provides below 3% of the RDA for males and females). In contrast, it provides significant contents of manganese, copper, zinc, iron and phosphorus. This research has shown that teff is a contributor to the RDA and AI for females for minerals and elements in the following order: manganese (up to 378%) > copper (up to 278%) > zinc (up to 93%) ≥ magnesium (up to 79%) > iron (up to 65%) ≥ phosphorus (up to 60%) > and calcium (up to 27%); and for males: manganese (up to 296%) > copper (up to 278%) > iron (up to 146%) > zinc (up to 67%) ≥ phosphorus (up to 60%) ≥ magnesium (up to 60%) > and calcium (up to 27%). Previously, data regarding dietary intake levels of minerals and essential trace elements obtained from teff has been limited. So far, teff is known to be a valuable source of calcium, iron, manganese and zinc [1,5]. Based on the current study’s results, teff may be recommended as a significant source of copper and phosphorus as well. The low RDA of copper is rare and may be caused by interfering factors which have an impact on its bioavailability, such as iron deficiency or excessive zinc ingestion. Furthermore, anti-nutrient phytic acid influences a bioavailability of zinc, iron and manganese [18]. Considering the fact that more than seventy enzymes in the human body employ zinc as an essential co-factor, teff, especially from the USA, may be recommended as a value-added cereal. Its contribution to the RDA for calcium is significant, especially if compared with rice which only contributes 0.6% of the RDA for calcium [18].

The contribution of teff to aluminium, cadmium, tin and mercury intakes was calculated as the weekly intake based on the PTWIs; and its contribution to cadmium was calculated as the monthly intake based on the PTMI set by the JECFA [11,12,13,14]. The PTWIs and PTMIs are displayed in Table 3. The estimated teff contribution to the PTWI for aluminium is 6% for males and 7% for females; its contribution to the PTMI for cadmium is 0.7% for females and 0.6% for males. Concerning mercury, the PTWI is lower than 0.8% for females and 0.7% for males. The PTWI for tin is lower than 0.001% for both males and females. The PTWIs for arsenic and lead were withdrawn in 2011 [12,29]. The PTWI (or PTMI) expresses the long-term exposure risk for contaminants that may accumulate in the human body. This research has proven that a serving portion of teff grains does not significantly contribute to the PTWIs (or PTMIs) for aluminium, cadmium, mercury and lead.

## 4. Conclusions

This study provided data on the minerals in white and brown teff grains that may be suitable as markers of essential, trace and risk elements. It also discussed nutritional dietary intakes and compared the amount of trace elements in teff with other cereal species.

Teff grains are rich in manganese, copper, phosphorus, iron, manganese, calcium and zinc. Their daily intakes have been calculated by applying the RDAs or AIs. It should be highlighted that teff originating in the USA contained substantial amounts of zinc. An arsenic concentration higher than the average arsenic content in cereals consumed in the EU was recorded in brown teff from Bolivia harvested in 2017. The contribution of teff to the PTWIs or PTMIs for metals was within the limits set by the FAO/WHO. Therefore, average teff consumption does not pose a health risk.

The interest in producing teff outside Ethiopia and providing it to food markets will continue to grow. As this study has shown, nutrient-dense teff grains may be considered to be a valuable source of minerals and other substances with desirable health benefits. Teff should be included into a common diet as it can improve the health of the general population.

## Figures and Tables

**Table 1:** Content of selected minerals and trace elements in brown and white teff grains evaluated by Inductively coupled plasma mass spectrometry (ICP-MS).

Analyte	Brown Teff		Brown Teff		Brown Teff	White Teff		White Teff	
	Bolivia		USA		EU	Bolivia		USA	
	I.	II.	I.	II.	I.	I.	II.	I.	II.
mg/kg									
^23^Na	185 ± 2 ^a^	159 ± 3 ^b,f^	130 ± 2 ^c,h^	135 ± 2 ^d,i^	124 ± 2 ^e^	161 ± 3 ^f^	155 ± 2 ^g^	131 ± 2 ^h^	137 ± 3 ^i^
^24^Mg	2090 ± 20 ^a^	1870 ± 20 ^b^	2530 ± 25 ^c^	2420 ± 20 ^d^	2080 ± 30 ^a^	1850 ± 30 ^b^	1760 ± 10 ^e^	2300 ± 20 ^f^	2400 ± 30 ^d^
^27^Al	10.8 ± 0.1 ^a^	13.4 ± 0.1 ^b^	12.1 ± 0.1 ^c^	11.5 ± 0.1 ^d^	9.11 ± 0.10 ^e^	5.42 ± 0.10 ^f^	5.92 ± 0.10 ^g^	8.13 ± 0.20 ^h^	7.70 ± 0.20 ^i^
^31^P	4070 ± 20 ^a^	4180 ± 30 ^b^	4160 ± 30 ^b^	4050 ± 20 ^a^	3740 ± 30 ^c^	3850 ± 20 ^d^	3770 ± 30 ^c^	3950 ± 30 ^e^	3930 ± 30 ^f^
^39^K	4290 ± 20 ^a^	4730 ± 30 ^b^	2750 ± 20 ^c^	2970 ± 20 ^d^	2980 ± 20 ^d^	2970 ± 10 ^d^	2970 ± 20 ^d^	3190 ± 20 ^e^	3080 ± 20 ^f^
^44^Ca	1980 ± 20 ^a^	2090 ± 10 ^b^	2640 ± 20 ^c^	2650 ± 40 ^c^	2310 ± 20 ^d^	1650 ± 20 ^e^	1760 ± 20 ^f^	2630 ± 30 ^c^	2420 ± 20 ^d^
^55^Mn	40.1 ± 1.0 ^a^	68.4 ± 2.4 ^b^	19.1 ± 1.2 ^c,d,h,i^	17.1 ± 1.3 ^d,h,i^	23.5 ± 1.1 ^e^	50.2 ± 1.4 ^f^	59.7 ± 2.1 ^g^	17.4 ± 1.0 ^h,i^	17.8 ± 1.2 ^i^
^57^Fe	96.4 ± 2.7a	94.1 ± 2.8 ^a^	115.0 ± 2.0 ^b,c,g,h^	112.0 ± 3.0 ^c,g^	89.4 ± 1.5 ^d,e^	87.5 ± 1.4 ^e^	83.6 ± 1.6 ^f^	111 ± 3.0 ^g^	117 ± 2.0 ^h^
^63^Cu	6.21 ± 0.20 ^a^	6.12 ± 0.10 ^a^	20.1 ± 1.2 ^b,d^	23.7 ± 2.1 ^c,g,h^	19.6 ± 1.5 ^d^	7.04 ± 0.20 ^e^	6.61 ± 0.20 ^f^	22.8 ± 0.8 ^g^	25.3 ± 1.0 ^h^
^66^Zn	24.7 ± 0.7 ^a^	23.5 ± 0.8 ^a,e^	68.5 ± 1.8 ^b,f^	74.2 ± 2.0 ^c,g^	34.7 ± 1.1 ^d^	23.6 ± 0.9 ^a^	21.5 ± 0.5 ^e^	66.9 ± 1.7 ^f^	73.0 ± 1.4 ^g^
^137^Ba	7.02 ± 0.10 ^a^	2.21 ± 0.10 ^b^	5.33 ± 0.10 ^c^	4.42 ± 0.08 ^d^	2.22 ± 0.10 ^b^	3.61 ± 0.10 ^e^	3.84 ± 0.10 ^f^	4.40 ± 0.10 ^d^	4.41 ± 0.10 ^d^
µg/kg									
^7^Li	15.4 ± 0.3 ^a^	39.1 ± 0.6 ^b^	166 ± 2 ^c^	182 ± 2 ^d^	34.7 ± 1.6 ^e^	24.7 ± 1.3 ^f^	21.5 ± 1.1 ^g^	155 ± 2 ^h^	149 ± 2 ^i^
^9^Be	2.30 ± 0.08 ^a^	12.2 ± 0.1 ^b^	5.72 ± 0.08 ^c^	5.31 ± 0.05 ^d^	3.73 ± 0.10 ^e^	6.71 ± 0.10 ^f^	6.23 ± 0.10 ^g^	15.5 ± 0.2 ^h^	11.6 ± 0.1 ^i^
^45^Sc	53.5 ± 1.2 ^a^	95.1 ± 1.0 ^b^	11.3 ± 0.1 ^c^	12.6 ± 0.1 ^d^	13.5 ± 0.1 ^e^	41.3 ± 1.0 ^f^	42.6 ± 1.2 ^f^	13.3 ± 0.1 ^e^	10.5 ± 0.1 ^g^
^48^Ti	363 ± 8 ^a^	407 ± 9 ^b^	847 ± 9 ^c^	723 ± 9 ^d^	505 ± 9 ^e^	463 ± 8 ^f^	439 ± 9 ^g^	728 ± 7 ^d^	683 ± 7 ^h^
^52^Cr	12.0 ± 0.2 ^a^	23.2 ± 0.7 ^b^	5.32 ± 0.05 ^c^	6.22 ± 0.07 ^d^	2.33 ± 0.04 ^e^	12.4 ± 0.3 ^a^	10.5 ± 0.2 ^f^	9.04 ± 0.06 ^g^	8.61 ± 0.10 ^h^
^59^Co	29.1 ± 0.6 ^a^	31.9 ± 0.5 ^b^	39.0 ± 0.9 ^c^	35.8 ± 1.0 ^d^	25.9 ± 1.1 ^e^	34.5 ± 1.1 ^d^	36.2 ± 1.2 ^d^	42.1 ± 0.8 ^f^	34.7 ± 0.6 ^d^
^60^Ni	295 ± 3 ^a^	251 ± 2 ^b^	403 ± 4 ^c^	377 ± 4 ^d^	412 ± 3 ^e^	276 ± 3 ^f^	226 ± 3 ^g^	477 ± 5 ^h^	438 ± 3 ^i^
^71^Ga	2.30 ± 0.10 ^a^	1.22 ± 0.10 ^b,c^	1.11 ± 0.08 ^b^	1.11 ± 0.10 ^b^	1.12 ± 0.10 ^b^	1.23 ± 0.10 ^b,c^	1.21 ± 0.10 ^b,c^	1.31 ± 0.10 ^b,c^	1.21 ± 0.10 ^b,c^
^75^As	45.5 ± 1.0 ^a^	65.9 ± 1.2 ^b^	30.1 ± 0.6 ^c,g^	34.3 ± 0.5 ^d,f,g^	15.6 ± 0.3 ^e^	34.7 ± 1.0 ^f^	28.6 ± 0.7 ^g^	31.4 ± 0.7 ^h^	23.3 ± 0.5 ^i^
^77^Se	10.5 ± 0.1 ^a^	9.81 ± 0.10 ^b^	15.7 ± 0.3 ^c^	17.5 ± 0.3 ^d^	13.7 ± 0.3 ^e^	9.30 ± 0.10 ^f^	8.61 ± 0.10 ^g^	17.9 ± 0.3 ^h^	19.3 ± 0.2 ^i^
^88^Sr	527 ± 4 ^a^	448 ± 4 ^b^	257 ± 3 ^c^	281 ± 3 ^d^	427 ± 3 ^e^	317 ± 4 ^f^	283 ± 3 ^d^	215 ± 3 ^g^	225 ± 2 ^h^
^89^Y	30.0 ± 2.2 ^a,c^	27.6 ± 2.2 ^a^	30.2 ± 3.0 ^a,c^	18.5 ± 1.1 ^b,e^	34.7 ± 1.0 ^c^	25.7 ± 2.5 ^a^	31.4 ± 1.1 ^c^	14.5 ± 1.1 ^d^	17.9 ± 1.1 ^e^
^107^Ag	13.4 ± 0.2 ^a^	20.2 ± 0.3 ^b^	19.2 ± 0.5 ^c^	28.4 ± 1.0 ^d^	34.9 ± 0.9 ^e^	15.8 ± 0.4 ^f^	17.1 ± 0.5 ^g^	33.8 ± 0.6 ^h^	28.7 ± 0.7 ^d^
^111^Cd	1.11 ± 0.10 ^a^	1.22 ± 0.10 ^a,d^	3.72 ± 0.08 ^b^	3.83 ± 0.10 ^b^	1.73 ± 0.10 ^c^	1.22 ± 0.10 ^a,d^	1.31 ± 0.10 ^d^	3.12 ± 0.10 ^e^	3.31 ± 0.10 ^f^
^118^Sn	0.71 ± 0.01 ^a^	1.72 ± 0.02 ^b^	0.61 ± 0.02 ^a,e^	0.72 ± 0.03 ^a^	0.62 ± 0.08 ^a,e^	0.90 ± 0.01 ^c^	1.24 ± 0.01 ^d^	0.63 ± 0.02 ^a,e^	0.50 ± 0.01 ^e^
^133^Cs	25.8 ± 0.4 ^a^	8.44 ± 0.01 ^b^	30.0 ± 1.0 ^c^	40.6 ± 1.1 ^d^	43.2 ± 1.0 ^e^	14.2 ± 0.3 ^f^	17.7 ± 0.2 ^g^	72.2 ± 1.2 ^h^	60.3 ± 1.0 ^i^
^140^Ce	23.8 ± 0.5 ^a^	41.7 ± 1.0 ^b^	49.6 ± 1.1 ^c^	41.0 ± 1.2 ^d^	18.1 ± 0.4 ^e^	26.9 ± 0.3 ^f^	28.4 ± 0.2 ^g^	20.4 ± 0.3 ^h^	24.8 ± 0.3 ^i^
^159^Tb	0.61 ± 0.01 ^a,c^	2.12 ± 0.01 ^b^	0.62 ± 0.01 ^a,c^	0.63 ± 0.02 ^a,c^	0.51 ± 0.01 ^a^	0.63 ± 0.02 ^a,c^	0.71 ± 0.02 ^c^	1.81 ± 0.02 ^d^	1.32 ± 0.03 ^e^
^165^Ho	1.00 ± 0.10 ^a,c^	1.81 ± 0.10 ^b^	1.10 ± 0.10 ^a^	1.02 ± 0.10 ^a,c^	0.92 ± 0.10 ^c^	1.42 ± 0.10 ^d^	1.72 ± 0.10 ^b^	2.31 ± 0.10 ^e^	2.40 ± 0.10 ^e^
^181^Ta	0.20 ± 0.10 ^a^	0.21 ± 0.10 ^a^	0.20 ± 0.10 ^a^	0.20 ± 0.10 ^a^	0.27 ± 0.10 ^a^	ND	ND	ND	ND
^202^Hg	2.31 ± 0.01 ^a^	1.42 ± 0.02 ^b^	1.42 ± 0.01 ^b^	1.83 ± 0.03 ^c^	1.82 ± 0.02 ^c^	1.52 ± 0.03 ^b,d^	1.60 ± 0.05 ^d^	3.11 ± 0.01 ^e^	2.91 ± 0.01 ^f^
^205^Tl	2.20 ± 0.01 ^a^	2.41 ± 0.01 ^b^	1.10 ± 0.02 ^c^	1.11 ± 0.02 ^c^	1.33 ± 0.01 ^d^	2.42 ± 0.02 ^b^	2.00 ± 0.01 ^e^	2.12 ± 0.01 ^e^	2.11 ± 0.01 ^e^
^208^Pb	0.63 ± 0.01 ^a^	1.63 ± 0.05 ^b^	1.14 ± 0.01 ^c^	1.42 ± 0.03 ^b,d^	1.00 ± 0.01 ^c^	1.11 ± 0.02 ^c^	1.33 ± 0.02 ^d^	1.11 ± 0.01 ^c^	0.84 ± 0.01 ^e^
^209^Bi	6.10 ± 0.01 ^a^	6.82 ± 0.02 ^b^	3.14 ± 0.02 ^c^	2.90 ± 0.05 ^d^	1.04 ± 0.02 ^e^	3.44 ± 0.03 ^f^	3.14 ± 0.1 ^c^	2.34 ± 0.03 ^g^	2.41 ± 0.05 ^g^
^238^U	5.04 ± 0.03 ^a^	9.71 ± 0.02 ^b^	2.04 ± 0.01 ^a^	2.22 ± 0.01 ^c^	2.71 ± 0.02 ^d^	1.62 ± 0.01 ^e^	2.14 ± 0.03 ^c^	4.04 ± 0.05 ^f^	3.98 ± 0.05 ^f^

Results are presented in fresh weight as means ± SDs (*n* = 5). Means within a line with at least one identical superscript do not differ significantly (*p* ≥ 0.05); ND: not detectable; I., grains harvested in 2016; II., grains harvested in 2017.

**Table 2:** Daily intake estimations for essential minerals in white and brown teff grains.

Analyte	Range (mg/kg)	Daily Intake (mg/Day)	RDA or AI * (F) (mg/Day)	RDA or AI * (M) (mg/Day)	RDA or AI * (F) (%)	RDA or AI * (M) (%)
Mg	1760–2530	176–253	320	420	55–79	42–60
P	3740–4180	374–418	700	700	53–60	53–60
K	2750–4730	275–473	4700 *	4700 *	6–10 *	6–10 *
Ca	1650–2650	165–265	1000	1000	17–27	17–27
Na	124–185	12.4–18.5	1500 *	1500 *	1 *	1 *
Mn	17.1–68.4	1.7–6.8	1.8 *	2.3 *	94–378 *	74–296 *
Fe	83.6–117.0	8.4–11.7	18	8	47–65	105–146
Cu	6.12–25.30	0.6–2.5	0.9	0.9	67–278	67–278
Zn	21.5–74.2	2.2–7.4	8	11	28–93	20–67
Cr	0.002–0.023	0.0002–0.0023	0.025 *	0.035 *	1–9 *	1–7 *
Se	0.009–0.019	0.0009–0.0019	0.055	0.055	2–3	2–3

AI *: Adequate intake is followed by an asterisk (*); RDA: Recommended Daily Allowance is written in ordinary type without an asterisk; M: male 31−50 years old; F: female 31−50 years old. A serving size of teff was set to 100 g.

**Table 3:** Intake estimations for toxic elements in white and brown teff grains.

Analyte	Range (µg/kg)	Daily Intake (µg/Day)	Weekly/Monthly * Intake (µg)	PTWI PTMI * (µg/kg)	PTWI PTMI * (F, 65 kg), (%)	PTWI PTMI * (M, 80 kg), (%)
Al	5420–13,400	542–1340	3790–9380	2000	3–7	2–6
Cd	1.11–3.83	0.111–0.383	3.33–11.49 *	25 *	<0.7 *	<0.6 *
Sn	0.50–1.72	0.050–0.172	0.35–1.20	14000	<0.001	<0.001
Hg	1.42–3.11	0.142–0.311	0.99–2.18	4	<0.8	<0.7

PTWI: Provisional tolerable weekly intake; PTMI: Provisional tolerable monthly intake is followed by an asterisk (*); M: male, 80 kg; F: female, 65 kg. A serving size of teff was set as 100 g.
